# Radiomics-Based Detection of Germ Cell Neoplasia In Situ Using Volumetric ADC and FA Histogram Features: A Retrospective Study

**DOI:** 10.3390/cancers17193220

**Published:** 2025-10-02

**Authors:** Maria-Veatriki Christodoulou, Ourania Pappa, Loukas Astrakas, Evangeli Lampri, Thanos Paliouras, Nikolaos Sofikitis, Maria I. Argyropoulou, Athina C. Tsili

**Affiliations:** 1 Department of Clinical Radiology, Faculty of Medicine, School of Health Sciences, University of Ioannina, 45110 Ioannina, Greece; m.christodoulou@uoi.gr (M.-V.C.); o.pappa@uoi.gr (O.P.); margyrop@uoi.gr (M.I.A.); 2 Department of Medical Physics, Faculty of Medicine, School of Health Sciences, University of Ioannina, 45110 Ioannina, Greece; astrakas@uoi.gr; 3 Department of Pathology, Faculty of Medicine, School of Health Sciences, University of Ioannina, 45110 Ioannina, Greece; elampri@uoi.gr; 4 Department of Urology, Faculty of Medicine, School of Health Sciences, University of Ioannina, 45110 Ioannina, Greece; a.paliouras@uoi.gr (T.P.); nsofikit@uoi.gr (N.S.)

**Keywords:** carcinoma in situ, diffusion magnetic resonance imaging, diffusion tensor imaging, radiomics, testicular tumors

## Abstract

Background/Objectives: Germ Cell Neoplasia In Situ (GCNIS) is considered the precursor lesion for the majority of testicular germ cell tumors (TGCTs). The aim of this study was to evaluate whether first-order radiomics features derived from volumetric diffusion tensor imaging (DTI) metrics—specifically apparent diffusion coefficient (ADC) and fractional anisotropy (FA) histogram parameters—can detect GCNIS. Methods: This study included 15 men with TGCTs and 10 controls. All participants underwent scrotal MRI, including DTI. Volumetric ADC and FA histogram metrics were calculated for the following tissues: group 1, TGCT; group 2: testicular parenchyma adjacent to tumor, histologically positive for GCNIS; and group 3, normal testis. Non-parametric statistics were used to assess differences in ADC and FA histogram parameters among the three groups. Pearson’s correlation analysis was followed by ordinal regression analysis to identify key predictive histogram parameters. Results: Widespread distributional differences (*p* < 0.05) were observed for many ADC and FA variables, with both TGCTs and GCNIS showing significant divergence from normal testes. Among the ADC statistics, the 10th percentile and skewness (*p* = 0.042), range (*p* = 0.023), interquartile range (*p* = 0.021), total energy (*p* = 0.033), entropy and kurtosis (*p* = 0.027) proved the most significant predictors for tissue classification. FA_energy (*p* = 0.039) was the most significant fingerprint of the carcinogenesis among the FA metrics. These parameters correctly characterized 88.8% of TGCTs, 87.5% of GCNIS tissues and 100% of normal testes. Conclusion: Radiomics features derived from volumetric ADC and FA histograms have promising potential to differentiate TGCTs, GCNIS, and normal testicular tissue, aiding early detection and characterization of pre-cancerous lesions.

## Simple Summary

Germ Cell Neoplasia In Situ (GCNIS) is a precursor to most testicular germ cell tumors (TGCTs), and its non-invasive detection could greatly enhance early diagnosis and management. This retrospective study assessed the utility of first-order radiomics features derived from volumetric apparent diffusion coefficient (ADC) and fractional anisotropy (FA) histograms in identifying GCNIS. The study cohort included 16 men with histologically confirmed TGCTs and 10 healthy controls. Quantitative analysis revealed significant differences in several volumetric ADC and FA metrics among TGCT, GCNIS-positive testicular tissue, and normal testicular parenchyma. These imaging-derived parameters demonstrated high diagnostic accuracy, correctly classifying 88.8% of TGCTs, 87.5% of GCNIS-positive tissues, and 100% of normal testicular tissues. These findings suggest that radiomics features extracted from volumetric ADC and FA histogram analysis may serve as valuable non-invasive biomarkers for the early detection of GCNIS and provide insights into testicular tumorigenesis.

## 1. Introduction

Testicular cancer represents approximately 1% of all adult malignancies and accounts for about 5% of urological tumors [1,2]. Its incidence is on the rise, particularly in industrialized countries [1,2,3]. The vast majority (95%) of testicular cancers are testicular germ cell tumors (TGCTs), most of which originate from Germ Cell Neoplasia in Situ (GCNIS) [1,4,5]. Post-pubertal TGCTs originating from GCNIS are further classified into two main categories based on developmental origin, and epigenetic characteristics: pure seminomas and non-seminomatous germ cell tumors (NSGCTs). NSGCTs include embryonal carcinoma, yolk sac tumor, choriocarcinoma, and post-pubertal teratoma [1,4,5].

Germ Cell Neoplasia In Situ is present in the contralateral testis in up to 9% of men with TGCTs. If left untreated, GCNIS progresses to invasive cancer in approximately 50% of patients within 5 years and in 70% within 7 years [1,6,7,8,9,10]. However, recommendations regarding routine biopsy of the contralateral testis in all patients with TGCTs remain controversial. This is due to the relatively low incidence of contralateral GCNIS and metachronous testicular tumours, the potential morbidity associated with GCNIS treatment, and the fact that most metachronous tumours are detected at an early stage. Currently, contralateral testicular biopsy should be considered in men at increased risk for GCNIS, including those with testicular atrophy, a history of cryptorchidism, or age under 40 years [1,6,7,11]. Non-invasive identification of GCNIS may provide an opportunity for early therapeutic planning before invasive TGCT develops.

Published data have suggested the use of conventional sonography and scrotal MRI for detecting GCNIS; however, their clinical utility has not been substantiated [12,13,14,15,16]. In a retrospective study involving 26 histologically confirmed TGCTs, MRI with diffusion-weighted imaging (DWI) and measurements of the apparent diffusion coefficient (ADC) values were used to assess the potential for detecting GCNIS [16]. Diffusion-weighted imaging evaluates the random Brownian motion of water molecules, which is restricted in biological tissues by structures such as cell membranes, fibers, and macromolecules. The degree of this restriction is quantified using ADC values [17,18]. In this study, ADC measurements were obtained from three tissue types: TGCTs; adjacent non-tumoral tissue, positive for the presence of GCNIS on histology; and normal testicular parenchyma. Although ADC values were lower in TGCTs compared to normal testes, the technique did not reliably distinguish GCNIS from either invasive carcinoma or normal testicular tissue [16]. However, this study was based on the measurement of the mean ADC using a two-dimensional region of interest (ROI), manually placed in a single representative area of the lesion—a method susceptible to sampling errors and subjective bias. Moreover, mean ADC values may not adequately capture the heterogeneity of the entire lesion or subtle histologic alterations [16].

Volumetric ADC histogram analysis is a quantitative radiomics method that extracts and analyzes first-order radiomics features by summarizing the distribution of ADC values across an entire three-dimensional (3D) tissue volume. This approach effectively captures the biological heterogeneity of malignancies and is less prone to subjective bias and sampling errors [19,20,21,22,23,24,25,26,27,28]. Volumetric ADC-based histogram metrics by providing insights into tissue cellularity, heterogeneity, and microstructure have been reported useful in the characterization of testicular lesions and differentiation between seminomas and NSGCTs [26,27,28].

Diffusion Tensor Imaging (DTI), which is based on DWI, is an advanced MRI technique that evaluates not only the intensity of water diffusion in tissues but also its directionality. Molecular diffusion is inherently a 3D process and can occur with varying probabilities along different directions [29,30,31,32,33,34,35]. This anisotropic diffusion is particularly evident in highly organized tissues, such as the testis, which contains well-structured components like seminiferous tubules, septa, and blood vessels that are radially oriented toward the mediastinum testis [36]. DTI enables the quantification and visualization of diffusion anisotropy, offering insights into tissue microarchitecture and structural integrity. Two key parameters derived from DTI are the ADC, which reflects the overall magnitude of water diffusion, and the fractional anisotropy (FA), which represents the degree of directional dependence of diffusion [29,30,31,32,33,34,35,36]. Published data have reported the applications of DTI on testis imaging, including the differentiation between malignant and benign testicular mass lesions, and the assessment of male infertility [36,37,38,39,40,41,42].

The purpose of this study was to evaluate whether first-order radiomics features derived from volumetric DTI metrics—specifically ADC and FA histogram parameters—can detect Germ Cell Neoplasia in Situ.

## 2. Materials and Methods

### 2.1. Study Cohort

This is a single-center, retrospective study that included nineteen consecutive men with histologically confirmed TGCTs (age range, 20–49 years; mean age, 34.06 years), who underwent scrotal MRI from February 2018 to November 2024. Radical orchiectomy was performed in all patients in less than one week from the MRI examination.

This study was approved by our Institutional Review Board, and the Local Ethics Committee. The requirement for informed consent from patients was waived due to the retrospective nature of the study.

During the same period, 10 consecutive asymptomatic men who volunteered to participate in the study (age range, 21–39 years; mean age, 27.1 years) were used as controls.

### 2.2. MRI Protocol

All MRI examinations were performed on a 3.0 T magnet (Philips MR Systems, Ingenia CX), using a multichannel surface small extremities coil. Participants were placed into the MRI unit in the supine position, with the feet first. Both testes were positioned at a similar distance from the coil by placing a towel beneath them, and the penis was fixed to the lower anterior abdominal wall, using an adhesive tape. Conventional sequences included axial spin-echo T1-weighted imaging and fast spin-echo T2-weighted imaging (T2WI) in the three orthogonal planes.

Coronal DTI was performed during quiet breathing, using a fat-saturated single-shot spin-echo planar imaging sequence with b-values of 0 and 900 s/mm^2^, and 15 diffusion directions.

In patients with testicular tumors, subtracted dynamic contrast-enhanced (DCE) imaging was followed using a 3D fast field echo sequence. Coronal images were obtained before and after the intravenous administration of gadopentetate dimeglumine (0.2 mmol/kg), followed by a 20 mL flush of physiological saline. Subsequently, the pre-contrast dataset was subtracted from each of the seven post-contrast image sets on a slice-by-slice basis to generate subtracted DCE images.

Table 1 presents the detailed MRI protocol used in the study.

### 2.3. MRI Data Postprocessing

The ADC and FA maps were automatically generated on the Philips workstation using the DTI processing software (IntelliSpace Portal, Philips, Version 8.0.2.20820, MR Diffusion tool). The ADC and FA DICOM images were then converted into gzipped NIfTI-1.1 format (.nii.gz) using the *dcm2nii* tool from MRIcroGL (https://www.nitrc.org/projects/mricrogl/ accessed on 1 August 2025).

**Figure 1:**
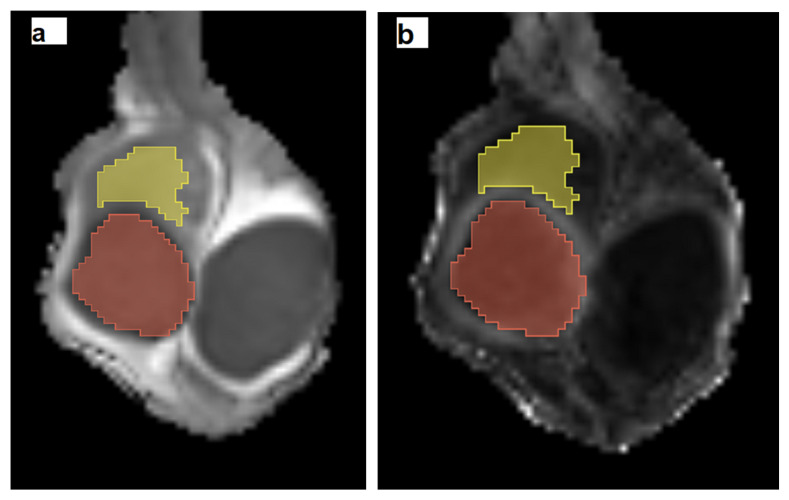
Three-dimensional masks of a testicular germ cell tumor (orange color) and the adjacent testicular parenchyma, histologically positive for Germ Cell Neoplasia In Situ (yellow color) are shown on coronal (**a**) ADC and (**b**) FA maps, created by the 3D Slicer software (https://3Dslicer.org, accessed on 1 August 2025). Volumes of interest (VOIs) were manually delineated within the tumor boundaries and the surrounding testicular tissue, confined by the tunica albuginea. T2-weighted images and dynamic contrast-enhanced MRI images were used as anatomical references for accurate VOI placement.

Volumetric histogram analysis of ADC and FA data was performed using the 3D Slicer software (https://3Dslicer.org, accessed on 1 August 2025). Three-dimensional masks were manually drawn on the ADC and FA maps for the following tissues: the testicular carcinoma, the non-tumoral part of the testis adjacent to the TGCT, histologically positive for the presence of GCNIS, and the entire testis in healthy controls. Segmentation was performed in consensus by three radiologists: A.C.T., a senior radiologist with 18 years of experience in scrotal MRI; M.V.C., a third-year radiology resident; and O.P., a fifth-year radiology resident. Specifically, in cases of TGCT, volumes of interest (VOIs) were manually delineated to encompass the entire tumor, while carefully remaining within the tumor boundaries. To minimize partial volume effects, the uppermost and lowermost slices of each lesion were excluded. In patients with multifocal tumors, only the largest lesion was segmented. Additional VOIs were carefully drawn in the testicular parenchyma adjacent to the tumor, representing the location of GCNIS, while carefully avoiding tumor margins and the tunica albuginea (Figure 1). T2-weighted images and DCE images were used as guidance. In healthy controls, VOIs were drawn to include the entire testis within the boundaries of the tunica albuginea, covering all relevant slices, with reference to T2WI.

Using the Informatics–Radiomics module in the 3D Slicer program, 18 first-order radiomics features were extracted from each ADC and FA map. These features included the 10th percentile, 90th percentile, maximum, mean, median, minimum, range, interquartile range (IQR), mean absolute deviation (MAD), robust mean absolute deviation (RMAD), root mean square (RMS), entropy, energy, total energy, skewness, kurtosis, uniformity, and variance. Table 2 provides brief descriptions of the first-order radiomic statistics derived from the volumetric ADC and FA maps [43].

### 2.4. Histologic Evaluation

Testicular specimens were fixed in 4% paraformaldehyde in PBS (pH 7.4) for 24 h prior to histologic examination. Samples were sequentially dehydrated in a graded ethanol series (70% to 100%), cleared in xylene, and embedded in paraffin wax. Serial transverse sections (4 μm thick) were cut using a microtome and subsequently stained with hematoxylin and eosin (H&E). Immunohistochemical staining was performed using a DakoCytomation Autostainer (DakoCytomation, Glostrup, Denmark). Briefly, 4-μm thick formalin-fixed, paraffin-embedded tissue sections were dewaxed in xylene and rehydrated through a graded ethanol series. Endogenous peroxidase activity was quenched by incubation with a peroxidase-blocking solution (DakoCytomation) according to the manufacturer’s instructions. Antigen retrieval was carried out by autoclaving the sections for 30 min in a pH 6.0 target retrieval solution (DakoCytomation).

**Figure 2:**
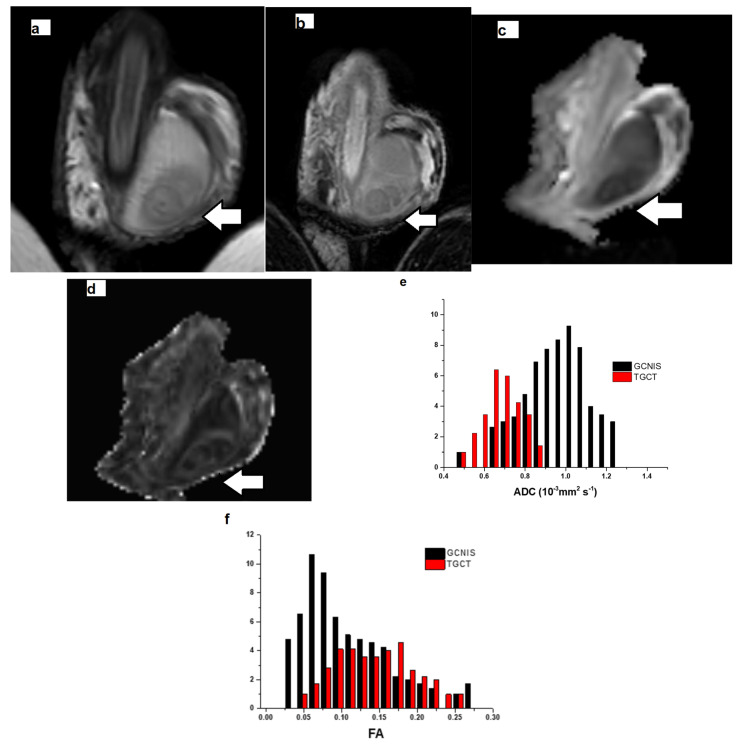

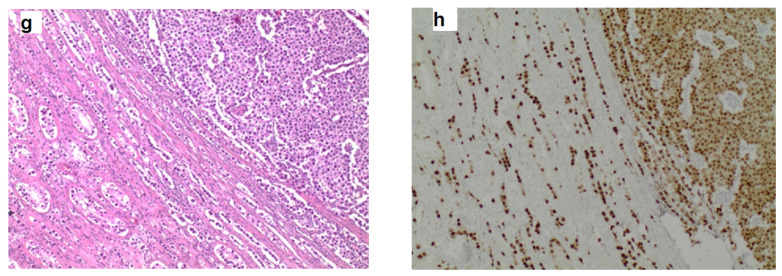
Left testicular seminoma in a 27-year-old man. The patient had right radical orchiectomy years ago due to seminoma. Coronal (**a**) T2-weighted and (**b**) subtracted dynamic contrast-enhanced (early phase) MRI images depict left intratesticular mass (arrow). The tumor appears mainly homogeneous and hypointense on T2-weighted image, with intratumoral septa, detected as bands of low T2 signal and strong enhancement after contrast medium administration. Coronal corresponding (**c**) ADC and (**d**) FA maps show testicular tumor (arrow) causing restricted diffusion –hypointense on ADC map– and increased anisotropy. Volumetric (**e**) ADC and (**f**) FA histograms of the testicular germ cell tumor (red color, TGCT) and the testicular tissue adjacent to the neoplasm, positive for the presence of Germ Cell Neoplasia In Situ (black color, GCNIS). The 10th percentile, range, interquartile range, entropy, total energy, skewness and kurtosis of ADC in seminoma were 0.05, 0.25, 0.04 (×10^−3^ mm^2^/s), 3.09, 34.72, 1.55, and 6.10, respectively. The 10th percentile, range, interquartile range, entropy, total energy, skewness and kurtosis of ADC in testicular tissue harboring GCNIS were 0.82, 0.75, 0.15 (×10^−3^ mm^2^/s), 7.97, 3564, −0.43, and 3.59, respectively. The FA energy in TGCT and tissue, positive for GCNIS was 2.70 and 60.48, respectively. (**g**) In the testicular parenchyma surrounding seminoma, intratubular germ cells consistent with GCNIS are identified (H&E ×100). (**h**) Immunohistochemical positivity for OCT3/4 was observed in GCNIS cells (×100).

**Figure 3:**
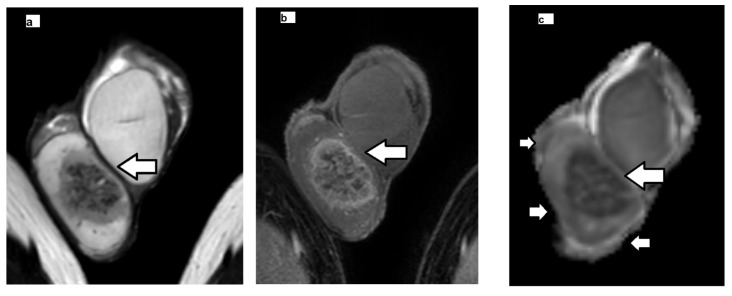

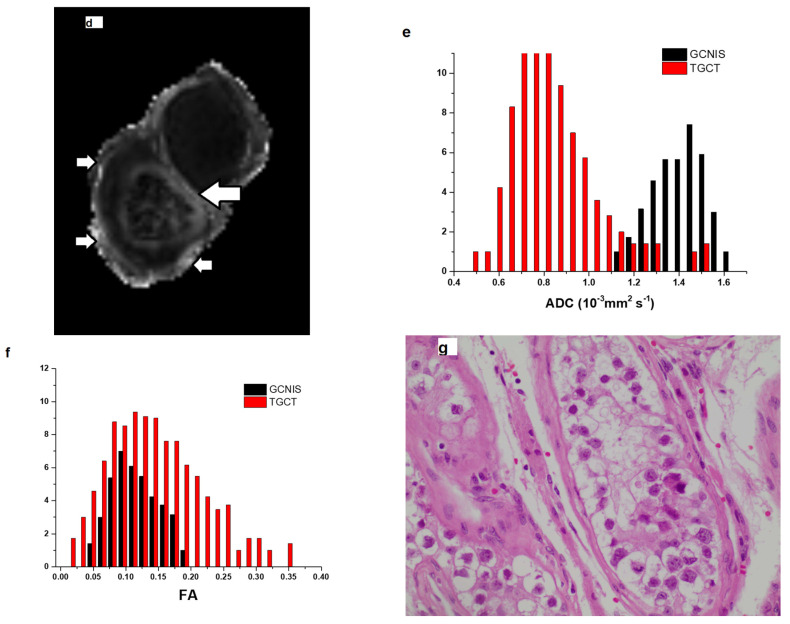
Embryonal carcinoma of the right testis in a 22-year-old man. Coronal (**a**) T2-weighted and (**b**) subtracted dynamic contrast-enhanced (early phase) MRI images depict right heterogeneous intratesticular tumor (arrow), enhancing avidly and inhomogeneously. Coronal corresponding (**c**) ADC and (**d**) FA maps show neoplasm (arrow) with restricted diffusion and increased anisotropy. Volumetric (**e**) ADC and (**f**) FA histogram analysis of the testicular germ cell tumor (red color, TGCT) and the testicular tissue surrounding the tumor, positive for the presence of Germ Cell Neoplasia In Situ (black color, GCNIS, small arrows (**c,d**)). The 10th percentile, range, interquartile range, entropy, total energy, skewness and kurtosis of ADC in embryonal carcinoma were 0.66, 1.02, 0.13 (×10^−3^ mm^2^/s), 8.3, 4130, 1.58, and 8.77, respectively. The 10th percentile, range, interquartile range, entropy, total energy, skewness and kurtosis of ADC in testicular tissue harboring GCNIS were 1.28, 0.46, 0.13 (×10^−3^ mm^2^/s), 7.29, 3577, −0.51, and 2.68, respectively. The FA energy in TGCT and tissue, positive for GCNIS was 14.76 and 2.65, respectively. (**g**) Intratubular atypical germ cells consistent with GCNIS, displaying prominent nucleoli and clear cytoplasm, aligned along the basement membrane of the seminiferous tubule (H&E ×400).

**Figure 4:**
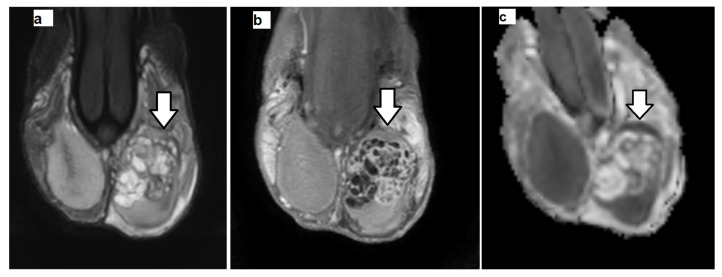

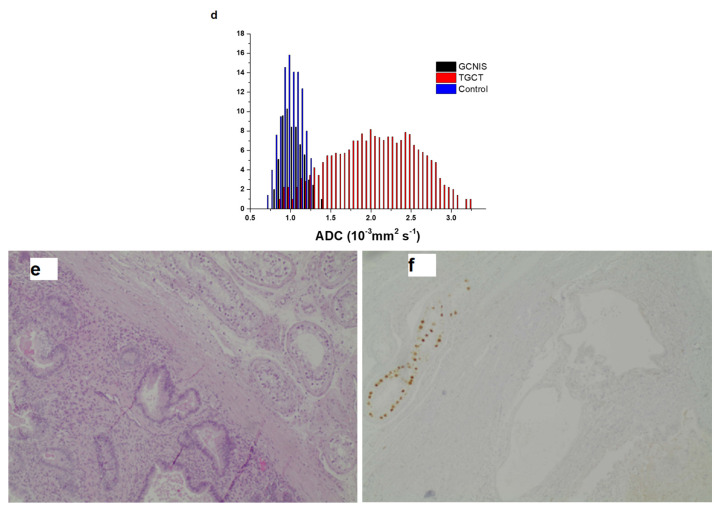
Non-seminomatous germ cell tumor (yolk sac tumor and mature cystic teratoma) of the left testis in a 20-year-old man. Coronal (**a**) T2-weighted and (**b**) subtracted dynamic contrast-enhanced (early phase) MRI images depict left intratesticular mass (arrow). The tumor appears heterogeneous on T2-weighted image, with strong, inhomogeneous contrast enhancement. Extensive areas of necrosis are detected within the neoplasm, hyperintense on T2-weighted image, and not enhancing after gadolinium administration. (**c**) Corresponding coronal ADC map shows testicular carcinoma (arrow), mainly hyperintense, due to its necrotic nature. (**d**) Volumetric ADC histograms of the testicular germ cell tumor (red color, TGCT) and the surrounding testicular tissue, positive for the presence of Germ Cell Neoplasia In Situ (black color, GCNIS). The histogram of the left normal testis (blue color) in a 22-year-old man is also shown. The 10th percentile, range, interquartile range, entropy, total energy, skewness and kurtosis of ADC in TGCT were 1.48, 2.42, 0.64 (×10^−3^ mm^2^/s), 9.79, 51744, −0.21, and 2.53, respectively. The 10th percentile, range, interquartile range, entropy, total energy, skewness and kurtosis of ADC in testicular tissue harboring GCNIS were 0.89, 0.58, 0.14 (×10^−6^ mm^2^/s), 7.93, 4332, 0.55, and 2.92, respectively. The 10th percentile, range, interquartile range, entropy, total energy, skewness and kurtosis of ADC in normal testicular tissue were 0.90, 0.57, 0.08 (×10^−3^ mm^2^/s), 4.00, 10985.3, 0.23, and 4.58, respectively. (**e**) Intratubular germ cells consistent with GCNIS are seen in the testicular parenchyma adjacent to the non-seminomatous tumor (H&E ×100). (**f**) GCNIS cells showed positive immunohistochemical staining for OCT3/4 (×100).

Histologically, GCNIS is characterized by large, atypical germ cells with prominent nucleoli, clear cytoplasm, and distinct cell borders, located within the seminiferous tubules, and typically aligned along the basement membrane (Figure 2, Figure 3 and Figure 4). Immunohistochemically, these neoplastic cells often exhibit strong nuclear expression of octamer-binding transcription factor 3/4 (OCT3/4)**,** placental alkaline phosphatase (PLAP), and c-KIT (CD117)**,** aiding significantly, especially in biopsies or limited specimens (Figure 2h and Figure 4f).

### 2.5. Statistical Analysis

Histogram parameters were classified into three tissue groups: Group 1—TGCT; Group 2—non-tumoral testicular parenchyma adjacent to the tumor, histologically positive for the presence of GCNIS; and Group 3—normal testicular parenchyma.

Statistical analysis was performed using IBM SPSS Statistics (version 26). Descriptive statistics, including median, minimum, and maximum values, were calculated for each group. Group differences in volumetric ADC and FA histogram features were assessed using the Kruskal–Wallis test. To correct for multiple comparisons, a Bonferroni adjustment was applied, resulting in a significance threshold of *p* < 0.00135. Post hoc pairwise comparisons between groups were subsequently performed, with statistical significance defined as *p* < 0.05 after adjustment.

Prior to multivariate analysis, highly correlated histogram metrics were excluded to reduce multicollinearity, which can negatively impact the stability of regression estimates. Redundant variables with a Pearson correlation coefficient greater than 0.9 (r > 0.9) were identified using the findCorrelation function from the caret package in R (version 4.2.1; R Core Team, Vienna, Austria).

The remaining variables were included as predictors in an ordinal logistic regression model to classify the ordered tissue groups (TGCT = 1, GCNIS = 2, controls = 3), reflecting increasing degrees of healthy tissue. The model employed a logit link function and was estimated using maximum likelihood. Model parameters were reported with 95% confidence intervals.

## 3. Results

### 3.1. Patient Population

Among the 19 men with TGCTs, one case was excluded from the analysis, due to the presence of artifacts on ADC and FA maps. The presence or absence of GCNIS was not included in the histologic report in two patients. Therefore, a total of 16 men with TGCTs, including 10 seminomas and six NSGCTs were finally assessed (Table 3). Histopathology reported the presence of GCNIS in the testicular parenchyma adjacent to carcinoma in 15 (93.75%) out of 16 TGCTs.

In the control group, testes were characterized as «normal» based on the absence of relevant clinical history and unremarkable MRI findings.

### 3.2. Histogram Metrics

The Kruskal–Wallis test revealed significant differences among the three tissue groups in 18 out of 36 radiomic features after applying the Bonferroni correction. Specifically, for the ADC-based volumetric histogram features, significant differences were found in 10th percentile, minimum, entropy, energy, total energy, and uniformity. Significant differences were observed in multiple FA-based volumetric histogram statistics, including 10th percentile, 90th percentile, mean, median, minimum, IQR, MAD, RMAD, RMS, entropy, uniformity, and variance.

**Figure 5:**
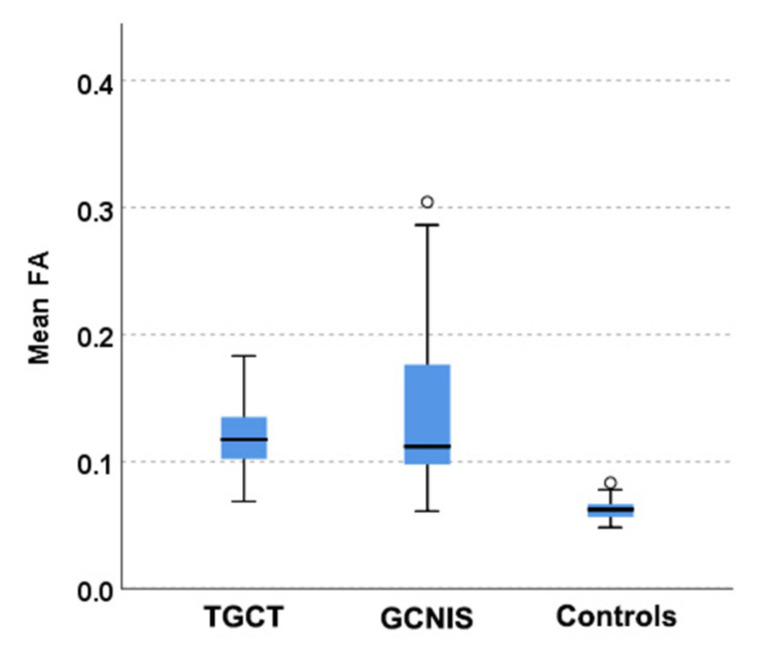
Boxplots showing distributions of mean FA in three tissue types: testicular germ cell tumor (TGCT), testicular parenchyma adjacent to tumor, histologically positive for the presence of germ cell neoplasia in situ (GCNIS), and normal testis. Post hoc pairwise comparisons with Bonferroni correction revealed significant differences between controls and both TGCTs and tissues with GCNIS (*p* < 0.001), but no differences between invasive and pre-invasive germ cell neoplasia (*p* = 0.839).

Groups 1 (TGCT) and 2 (non-tumoral testicular tissue adjacent to the tumor, histologically positive for GCNIS) showed substantial differences compared to group 3 (normal testicular tissue). For the ADC histogram metrics, entropy and energy had higher values in groups 1 and 2 compared to group 3. Conversely, total energy and uniformity were significantly reduced in TGCT and GCNIS-positive tissue when compared to the control group. For the FA histogram metrics, groups 1 and 2 demonstrated higher values for the 10th percentile, 90th percentile, mean, median, minimum, IQR, MAD, RMAD, RMS, entropy, and variance compared to group 3. In contrast, FA uniformity was decreased in both TGCT and GCNIS-positive tissue compared to normal testes. A summary of the descriptive statistics is provided in Table 4. A representative example of pairwise comparison is given in Figure 5, illustrating the distribution of mean FA values across the three tissue types using box plots. Both TGCTs and GCNIS-positive tissues exhibited significantly higher FA values compared to normal testicular tissue (*p* < 0.001), while no significant difference was observed between TGCT and GCNIS (*p* = 0.839), suggesting overlapping microstructural alterations between pre-invasive and invasive testicular malignancies. These findings confirm that increased anisotropy is a common feature of neoplastic tissue and demonstrate that volumetric FA histogram measures can reliably differentiate neoplastic from normal testicular parenchyma.

Post hoc pairwise comparisons revealed widespread distributional differences across the three groups for nearly all variables. Specifically, both groups 1 and 2 showed significant differences from group 3 (*p* < 0.05). However, no differences were observed between groups 1 and 2 for most volumetric histogram metrics. The only exception was ADC minimum, where the pairwise comparison revealed a significant difference (*p* < 0.001), with an increase found in group 2 (testicular tissue, harboring GCNIS) compared to group 1 (TGCT).

**Figure 6:**
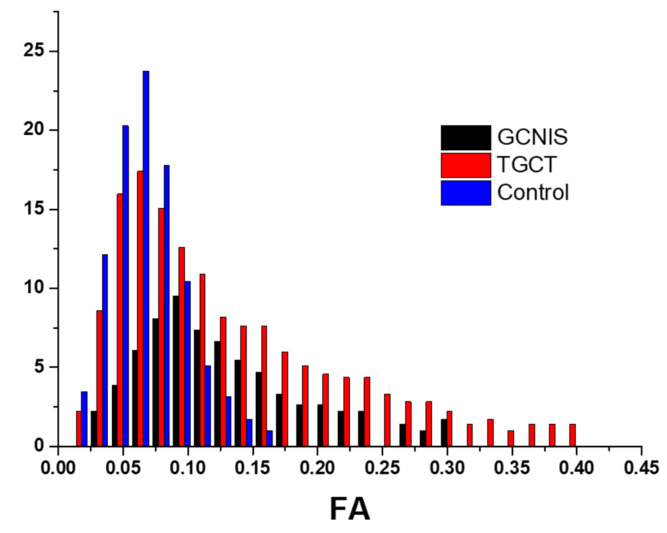
Volumetric FA histogram analysis of a left testicular germ cell tumor (seminoma, TGCT) depicted in red, in a 35-year-old man. The adjacent testicular parenchyma, positive for Germ Cell Neoplasia In Situ, is shown in black (GCNIS). For comparison, the FA distribution of normal testicular tissue from the left testis of a 30-year-old healthy male is shown in blue. The FA energy in TGCT, testicular tissue, positive for GCNIS, and normal testis was 18.78, 5.85, and 8.37, respectively. The comparison reveals a progressive rightward shift in the FA histograms—from normal tissue to GCNIS and TGCT. This pattern indicates increasing FA values, which likely reflect a gradual loss of microstructural organization and directional coherence within the tissue architecture.

Multicollinearity analysis eliminated highly correlated variables. The retained volumetric ADC variables included 10th percentile, range, IQR, entropy, total energy, skewness, kurtosis, and uniformity, while the retained FA variables included median, range, energy, skewness, kurtosis, and uniformity. The ordinal regression model demonstrated excellent fit (χ^2^ = 118.205, *p* < 0.001), explaining 88.8% of variance (Cox and Snell-R^2^ = 0.888). Specifically, it predicted the characterization of TGCTs in 88.8% of cases, the presence of GCNIS in the testicular parenchyma, adjacent to malignancy in 87.5% of cases, and the characterization of normal testes in all (100%) cases. The ordinal regression analysis demonstrated that tissue classification (from cancerous to precancerous and normal) was significantly associated with several first-order, volumetric histogram-derived diffusion metrics, generally supporting the hypothesis that cancerous tissue exhibits lower mean diffusivity (ADC) values with increased presence of outliers and higher FA values (Table 5). Among volumetric ADC histogram statistics, significant predictors of tissue classification included 10th percentile and skewness (*p* = 0.042), range (*p* = 0.023), IQR (*p* = 0.021), total energy (*p* = 0.033), entropy and kurtosis (*p* = 0.027) (Figure 2, Figure 3 and Figure 4). Among the FA histogram metrics, the energy (*p* = 0.039) proved the most significant fingerprint of the process of testicular carcinogenesis. Specifically, progression from normal testis to GCNIS and to frank malignancy was associated with a decrease in 10th percentile of ADC, a narrower ADC_range, and reduced total energy and skewness. Concurrently, higher IQR, entropy, and kurtosis were also significant predictors of malignancy, collectively indicating a less concentrated distribution of ADC values with more extreme observations. For FA, higher energy was significantly associated with the tumorigenesis process, consistent with the expectation of elevated FA in cancerous tissues (Figure 2, Figure 3, Figure 4 and Figure 6).

Figure 2 depicts a case of testicular seminoma, in which volumetric ADC histogram analysis revealed a narrow distribution with a lower peak of ADC values, indicating decreased intratumoral heterogeneity and restricted diffusion. These characteristics correlate with the histologic features of seminoma, which typically consists of large, uniform neoplastic cells and lacks areas of necrosis. In contrast, the ADC histogram of tissue with GCNIS showed a wider distribution and higher peak values, consistent with greater heterogeneity and less restricted diffusion. Similarly, the volumetric FA histogram of the seminoma showed a narrow distribution and higher peak FA values, reflecting a more homogeneous and disrupted microstructural architecture. Conversely, the FA histogram of tissue with GCNIS exhibited a broader distribution with a lower peak and positive skew, suggesting a less organized and more heterogeneous microstructural environment.

Histogram comparison of ADC values in Figure 3 (embryonal carcinoma) showed that tissue with GCNIS exhibited a narrower range and higher peak of ADC values—consistent with more homogeneous and less restricted diffusion—whereas TGCT displayed broader distributions with lower peak ADC values, higher IQR, and occasional high-value outliers, reflecting increased intratumoral heterogeneity and diffusion restriction. A similar pattern was observed in the volumetric FA histograms in the same case: CGNIS demonstrated a tight distribution with higher lower FA values, suggesting less organized microstructure, while embryonal carcinoma displayed a wider FA spread and higher peak values, consistent with organized but heterogeneous tissue microstructure.

## 4. Discussion

In our study, first-order radiomics features derived from volumetric DTI metrics—specifically, ADC and FA histogram parameters—were utilized to differentiate between three tissue types: TGCTs, testicular parenchyma histologically positive for GCNIS, and normal testes. Our preliminary observations revealed that many volumetric ADC and FA histogram metrics enabled differentiation not only between normal testes and TGCTs but also—more critically—between normal tissue and GCNIS-harboring parenchyma, demonstrating decreased diffusivity and increased anisotropy in both pre-invasive and invasive testicular malignancies.

While GCNIS foci are below the spatial resolution of conventional MRI, diffusion MRI is inherently sensitive to microstructural barriers at the cellular and sub-voxel level. Within the seminiferous tubules, water diffusion is restricted by cells, membranes, and the surrounding stroma—factors that can alter both ADC and FA values, even in the absence of a directly visible lesion. Our volumetric ADC and FA histogram metrics thus reflects parenchymal-level microstructural changes rather than discrete micronodules, supporting the notion that diffusion imaging may serve as a sensitive, non-invasive marker for pre-invasive testicular neoplasia.

Among the evaluated histogram-based features, several noninvasive biomarkers emerged that reflect testicular carcinogenesis, correctly characterizing 88.8% of TGCTs, 87.5% of GCNIS-positive tissues, and 100% of normal testes. With respect to ADC histogram parameters, a progressive decrease in 10th percentile, range, total energy, and skewness, and an increase in interquartile range, entropy, and kurtosis were observed along the spectrum from normal tissue to GCNIS to TGCT. For FA histogram metrics, an increase in energy emerged as a significant predictor of both GCNIS and invasive cancer.

First-order volumetric ADC and FA statistical parameters, derived from gray-level intensity histograms, quantitatively characterize the distribution of signal intensities within a defined VOI encompassing the entire tissue across all contiguous slices. Unlike previous methods that relied on mean ADC and FA values from manually drawn regions of interest on a single slice—approaches that are susceptible to outliers and highly dependent on ROI placement—this volumetric technique captures the full distribution of values within the entire tissue. As such, it more comprehensively reflects tumor heterogeneity, providing a more accurate representation of microstructural variations in both pre-invasive and invasive testicular malignancies. The technique enables the detection of intratumoral features such as heterogeneous cellularity, angiogenesis, extracellular stroma composition, and variable amounts of cystic components, hemorrhage, and necrosis. By minimizing sampling errors and reducing operator-dependent variability, this approach significantly enhances reproducibility, and diagnostic reliability [19,20,21,22,23,24,25,26,27,28].

A decrease in diffusion and an increase in anisotropy of TGCTs, compared to normal testes has already been reported on published studies using the mean ADC and FA values [16,36,37,44,45,46,47,48].

The ADC value reflects the degree of water molecule diffusion within tissues and is influenced by several factors, including tissue cellularity, fraction of extracellular space, and presence of diffusion barriers such as cell membranes and macromolecules [17,18]. In TGCTs, histological features such as increased cellularity, large and densely packed malignant cells, nuclear enlargement, irregular nuclear contour, prominent nucleoli, and lymphocytic infiltration contribute to restricted diffusion [4,5]. These characteristics lead to a decrease in ADC values in TGCTs [16,36,37,44,45,46,47,48].

The FA value derived from DTI quantifies the degree of directionality of water molecules diffusion in biological tissues. FA values range from 0 to 1. A value of 0 indicates isotropic diffusion, meaning that the random movement of water molecules is uniform in all directions. A value of 1 indicates completely anisotropic diffusion, meaning that diffusion occurs only in one axis, and molecular movement is restricted in all other directions [29,30]. High cellularity, dense packing of neoplastic cells, and aligned fibrous structures—such as a well-developed fibrous capsule surrounding the tumor or intratumoral fibrovascular septa—contribute to the anisotropic diffusion detected in TGCTs [4,5,36].

In the current report, significant alterations were observed in multiple volumetric histogram metrics derived from ADC and FA data, offering new diagnostic insights into TGCTs.

For ADC-derived metrics, TGCTs were associated with lower values in 10th percentile, minimum, total energy, and uniformity, alongside increased values in entropy, and energy. These alterations suggest greater heterogeneity and reduced tissue uniformity in invasive testicular tumors. For FA-derived metrics, TGCTs exhibited increased values across most statistical parameters, including the following: percentile-based metrics—such as 10th and 90th percentiles; central tendency metrics—such as mean, median, minimum, and root mean square; dispersion metrics—such as interquartile range, mean absolute deviation, and robust mean absolute deviation; and, histogram entropy and variance, alongside a decreased uniformity, reflecting heterogeneity and increased microstructural complexity within testicular malignancies.

Most notably, this study is the first in the literature to demonstrate that differentiation between tissue harboring GCNIS and normal testicular tissue is feasible, based on volumetric ADC and FA statistics. Significant distributional differences in many ADC and FA parameters were observed between histologically positive for GCNIS testicular tissues and normal testes, suggesting the effectiveness of fist-order radiomics in characterizing pre-invasive testicular tumors. Specifically, the volumetric ADC and FA alterations in GCNIS-positive regions were similar to those observed in TGCTs, suggesting reduced diffusivity and increased anisotropy in testicular tissues adjacent to TGCTs, where GCNIS is present.

Histologically, GCNIS is characterized by large, atypical germ cells with abundant clear or vacuolated cytoplasm, and enlarged, irregular nuclei featuring prominent nucleoli. These abnormal cells are confined within the seminiferous tubules, which usually show a deranged spermatogenesis. Additionally, these tubules often exhibit a thickened peritubular basement membrane, and a reduced diameter [49,50,51,52]. All the aforementioned histologic changes explain the restricted diffusion and the increased anisotropic movement detected through volumetric histogram analysis of DTI data in testicular tissues harboring GCNIS. Our findings suggest that first-order radiomics features derived from volumetric ADC and FA data could effectively differentiate cancerous from non-cancerous testicular tissues, and could serve as a non-invasive biomarker for predicting the presence of GCNIS.

The minimum ADC emerged as a metric to allow differentiation between testicular tissues harboring GCNIS and TGCTs in the current investigation. This parameter represents the lowest diffusivity value in the VOI, highlighting the most restricted diffusion areas, as it is in cases of high-grade or aggressive tumors [19,20,24,25,43,53,54,55,56,57,58]. Based on published data, the minimum ADC had the highest diagnostic performance in differentiating testicular malignancies from benign intratesticular lesions, with low values observed in cases of malignant tumors [26,27]. Interestingly, in our study the minimum ADC increased in testicular tissues adjacent to malignancies, positive for GCNIS compared to TGCTs. These findings are difficult to explain, and could be correlated to the coexisting impairment of spermatogenesis, often seen in the testicular parenchyma surrounding TGCTs [39,40,41]. Another possible explanation is related to the focal distribution of GCNIS in the testicular tissue adjacent to tumor, with some seminiferous tubules containing abundant GCNIS, coexisting with tubules without GCNIS [59,60].

Several diffusion metrics emerged as potential biomarkers of testicular carcinogenesis in this report. Notably, there was a progressive decrease in 10th percentile, range, total energy, and skewness of ADC when comparing normal testicular tissue to tissue with GCNIS and subsequently to overt malignancy. Conversely, the ADC interquartile range, entropy, and kurtosis progressively increased in testicular tissues positive for GCNIS and TGCTs, compared to normal testes. Similarly, an increase in FA_energy was also observed.

The 10th percentile of ADC represents the threshold below which 10% of voxel measurements within the VOI fall [43,53]. It serves as a sensitive marker for regions with high cellularity and restricted diffusion, characteristics often seen in malignancies. Decrease in 10th percentile of ADC frequently correlates with higher tumor grade and more aggressive behavior [24,25,26,27,28,54,55,56,57,58]. The 10th percentile of ADC proved a significant discriminator between seminomas and non-seminomatous neoplasms, in a retrospective study assessing the role of volumetric ADC histogram analysis in the histologic characterization of TGCTs. The study included 24 TGCTs, and the 10th percentile of ADC demonstrated 100% sensitivity, and 92.86% specificity in differentiating between seminomas and NSGCTs [28]. In our study, the decrease in 10th percentile of ADC observed in pre-invasive and invasive testicular tumors can be attributed to histologic alterations, such as increased cellularity, dense cellular packing, and reduced extracellular space [4,5].

The ADC range refers to the difference between the maximum and minimum ADC values within the VOI, providing information about the heterogeneity or variability [43,53]. Malignancies often exhibit a narrow ADC range due to their dense cellular structure, and the resulting homogeneity in diffusion restriction [55,61]. In the present investigation, this pattern was observed in both pre-invasive and invasive testicular malignancies. Another non-invasive biomarker of testicular carcinogenesis identified was the interquartile range, which reflects the variability of the middle 50% of ADC values within the VOI [43,53]. Malignant tissues typically exhibit a lower overall range but a higher IQR, indicating relatively uniform diffusion restriction at the macroscopic level, yet increased microstructural heterogeneity within the tumor. This inverse relationship between range and IQR suggests that testicular malignancies may appear globally homogeneous while being locally heterogeneous—a pattern with potential implications for tumor characterization, grading, and treatment planning. Our investigation confirmed both findings.

Total energy is a first-order radiomics feature that reflects both the magnitude and homogeneity of gray-level distribution in ADC values across the VOI [43,53]. Lower total energy indicates greater heterogeneity and reduced overall signal intensity [26,61,62,63,64]. Testicular germ cell tumors are histologically heterogeneous malignancies, characterized by significant variations in intratumoral cellularity, angiogenesis, and extracellular stroma. Seminomas often exhibit fibrovascular septa, while NSGCTs may show varying degrees of cystic degeneration, hemorrhage, and necrosis [4,5]. These histological features contribute to a reduction in total energy on volumetric ADC histograms. Additionally, testicular tissue adjacent to TGCTs also displays marked heterogeneity [65]. This includes, besides GCNIS, the presence of peritubular and intratubular lymphocytic infiltrates, intertubular fibrosis, and microliths—histological changes that further explain the observed decrease in total energy [65].

Among the FA-derived metrics, energy has proven to be a reliable factor in characterizing the process of tumorigenesis, with higher values consistent with the increase in anisotropy observed in testicular tumors [36].

Skewness measures the asymmetry of the distribution of ADC values relative to the mean [43,53]. Positive skewness—characterized by a rightward tail in the histogram—indicates a predominance of low ADC values, which is typically associated with high cellularity and has been commonly reported in malignant tumors [27,28]. A retrospective study of 45 intratesticular lesions found that ADC skewness was significantly higher in malignant lesions compared to benign ones [28]. However, our findings did not replicate these observations. In contrast, we observed negative skewness in both TGCTs and tissues containing GCNIS, indicating a leftward tail of the histogram, with a greater number of voxels exhibiting high ADC values, and reflecting the underlying histologic complexity characteristics of both tissue types.

This study also found a progressive increase in ADC entropy and kurtosis in both TGCTs, and testicular parenchyma, positive for GCNIS compared to normal testes. Entropy is a statistical measure of randomness or complexity in the ADC values distribution, and kurtosis measures how sharply peaked or flat the distribution of ADC values is [43,53]. High entropy is often linked with malignancy, due to intratumoral heterogeneity, as it was seen in the current investigation [66,67]. A high kurtosis means that more voxels cluster around a mean value, with some extreme low or high ADC values, and is often associated with neoplasms [43,53,67,68]. Tumoral heterogeneity both in pre-invasive and invasive testicular tumors results in an increase in kurtosis, creating a more peaked ADC distribution with extremely low ADC values (due to dense cellular areas) and possibly some higher ADC values (due to coexisting histologic findings, such as cystic or necrotic areas).

Germ Cell Neoplasia In Situ—previously referred to as carcinoma in situ, intratubular germ cell neoplasia, or testicular intraepithelial neoplasia—was first described in 1972 [69]. In the initial report, two infertile men were found to have abnormal seminiferous epithelium with atypical germ cells on testicular biopsy. Within 4.5 years following the biopsy, both patients developed embryonal carcinoma of the testis [69]. The detection of GCNIS is crucial for preventing the progression to invasive TGCTs. In patients with TGCT and a normal contralateral testis, the identification of GCNIS can guide preventive treatment strategies. Current management options for confirmed GCNIS include orchiectomy, low-dose radiotherapy, or active surveillance with regular US monitoring [6,7,8,9,10,70,71]. Given the invasive nature and associated morbidity of surgical biopsy—currently the gold standard for GCNIS detection—the development of reliable non-invasive diagnostic methods would represent a significant clinical advancement. Based on our preliminary data, radiomics-based features extracted from volumetric ADC and FA histogram analysis show strong potential for distinguishing between TGCTs, GCNIS, and normal testicular tissue. These findings may support early detection and improved characterization of pre-cancerous testicular lesions.

Several limitations of this study should be acknowledged. First, a major limitation of the current report is its single-center, retrospective design and the relatively small sample size. Given that radiomics analyses often involve high-dimensional data, a larger cohort would enhance the robustness, and generalizability of the statistical findings. Therefore, our results should be interpreted as preliminary, and hypothesis-generating rather than definitive. Nevertheless, despite these limitations, we observed consistent distributional differences in key volumetric ADC and FA histogram-derived features, even after applying corrections for multiple comparisons and multicollinearity. These findings suggest a strong biological signal that warrants further exploration. Future prospective, multicenter studies with larger number of participants are needed to validate and refine these radiomics-based biomarkers.

Second, all volumes of interest were manually segmented by consensus among three radiologists. Manual segmentation is time-consuming, subject to interobserver variability, and lacks standardization. The adoption of semi-automated or fully automated segmentation techniques could improve the reproducibility and scalability of future analyses. Third, the predictive model was trained and tested on the same dataset due to the absence of an independent validation cohort. This raises concerns about overfitting and limits the robustness of the model. External validation using a separate, prospectively collected dataset is essential. Fourth, the analysis was restricted to first-order radiomics features. While these features capture basic statistical properties of ADC and FA values, higher-order features could provide more detailed information about tissue microstructure and heterogeneity, potentially enhancing diagnostic performance. In addition, histological confirmation was not available for the control group.

Finally, in the present study, we prioritized ordinal regression modeling, as it more accurately captures the ordered biological progression from normal testis to GCNIS, and ultimately to TGCT. While Receiver Operating Characteristic (ROC) curve analysis is a widely adopted method for assessing the diagnostic performance of imaging-derived biomarkers, it primarily addresses binary classification tasks. Future studies involving large cohorts could incorporate ROC curve analyses to complement the ordinal approach by providing binary classification metrics (e.g., GCNIS vs. normal testes, TGCT vs. normal testes), thereby further supporting the clinical utility and translational potential of these radiomics features.

## 5. Conclusions

This study demonstrates the promising role of first-order radiomics features derived from volumetric DTI metrics—specifically, ADC and FA histogram parameters—in non-invasively differentiating between TGCTs, testicular parenchyma histologically positive for GCNIS, and normal testicular tissue. Unlike conventional DTI measurements that rely on mean ADC and FA values from manually defined regions of interest, our volumetric, histogram-based approach offers a more comprehensive and reproducible method by capturing the full distribution of signal intensities across the entire tissue volume. This technique enhances diagnostic accuracy by more effectively reflecting underlying histopathologic heterogeneity.

Our findings highlight several radiomics-derived biomarkers of testicular carcinogenesis. Notably, progressive changes in volumetric ADC histogram features—including decreased 10th percentile, range, total energy, and skewness, alongside increased IQR, entropy, and kurtosis—were observed along the spectrum from normal testicular tissue to GCNIS to TGCT. Volumetric FA-derived features, particularly increased energy, also emerged as significant indicators of neoplastic transformation. Importantly, this is the first study to show that radiomics-based volumetric DTI analysis can distinguish GCNIS-harboring tissue from normal testes, a critical step in early detection and risk stratification.

By enabling early detection of GCNIS and providing detailed characterization of TGCTs, volumetric radiomics analysis of DTI data may inform clinical decision-making, improve risk assessment, and reduce reliance on invasive diagnostic procedures such as surgical biopsy. Further prospective, multicenter studies with larger cohorts are warranted to validate these preliminary findings and to facilitate clinical translation into routine testicular cancer diagnostics.

## Figures and Tables

**Table 1:** MRI (3.0 T) protocol used for the assessment of testicular tumors.

Protocol	T1WI	T2WI	DTI	DCE-MRI
Sequences	Spin-Echo	Turbo Spin-Echo	Fat-Saturated Single-Shot Spin-Echo Planar Imaging	3D Fast Field Echo
plane	axial	axial, sagittal, coronal		coronal
TR (ms)	485	4000	coronal	5.5
TE (ms)	8	100–120	3.700	1.94–3.5
slice thickness (mm)	3	2–3	72	2
gap (mm)	0.3	0.2–0.3	4	0
matrix (mm)	172 × 168	180 × 256	0.4	200 × 200
FOV (mm)	170 × 170	240 × 270	128 × 87	160 × 177
NSA	1	1–2	160 × 80	1
b value (s/mm^2^)			2	
flip angle			0, 900	10^0^
dynamic scans				8
scan duration (min)	3	2.30–4.30	3	1 min each

(T1WI: T1-weighted imaging, T2WI: T2-weighted imaging, DTI: diffusion tensor imaging; DCE: dynamic contrast-enhanced, TR: time repetition; TE: time echo, FOV: field of view, NSA: number of signals averaged; 3D: three-dimensional).

**Table 2:** Description of the volumetric ADC and FA histogram metrics (Ref. [43]).

ADC/FA Histogram Metrics	Description
10th Percentile	The ADC/FA value below which 10% of all ADC/FA voxel values lie
90th Percentile	The ADC/FA value below which 90% of all ADC/FA voxel values lie
Maximum	The maximum ADC/FA value within the VOI
Mean	The average ADC/FA value within the VOI
Median	The ADC/FA value below which 50% of all ADC/FA voxel values lie
Minimum	The minimum ADC/FA value within the VOI
Range	Measures the difference between the maximum and minimum ADC/FA values within the VOI
IQR	Measures the spread of the distribution of ADC/FA values, defined as the difference between 75th and 25th percentile
MAD	Measures the average distance between each ADC/FA intensity value and the mean intensity of a VOI. Quantifies variability, but unlike variance or standard deviation, it does not square the deviations. It is less sensitive to outliers than variance or standard deviation
RMAD	Provides a more outlier-resistant measure of variability compared to standard MAD. The lowest and highest 10% of ADC/FA values are excluded
RMS	Represents the square root of the average of the squared intensity values in a VOI. Reflects both the mean ADC/FA values and their variability
Entropy	Measures the inherent randomness of the ADC/FA values within the VOI
Energy	Describes the uniformity of the distribution of ADC/FA voxel values within the VOI
Total Energy	Reflects the overall signal strength of ADC/FA voxel values within the VOI
Skewness	Measures the asymmetry of the distribution of ADC/FA values around the mean
Kurtosis	Measures the peakedness of ADC/FA values within the VOI
Uniformity	Quantifies the evenness of the intensity of ADC/FA values in the VOI. It is closely related to energy
Variance	Quantifies the dispersion of the intensity of ADC/FA values around the mean within the VOI

(VOI: volume of interest; IQR: interquartile range; MAD: mean absolute deviation; RMAD: robust mean absolute deviation; RMS: root mean square).

**Table 3:** Histologic diagnosis of testicular germ cell tumors (n = number).

Histologic Type	n
Seminomas	10
Non-seminomas	6
embryonal carcinoma	3
embryonal carcinoma, yolk sac tumor, teratoma	1
seminoma, embryonal carcinoma, teratoma	1
yolk scac tumor, teratoma	1

**Table 4:** Comparative analysis of volumetric ADC and FA-derived histogram metrics between the three tissue types: group 1—testicular germ cell tumor; group 2—non-tumoral testicular parenchyma adjacent to the malignancy, histologically positive for the presence of Germ Cell Neoplasia in Situ; and group 3—normal testes.

Groups	1 Median [Min, Max]	2 Median [Min, Max]	3 Median [Min, Max]	*p*
ADC-Derived Metrics (10^−3^ mm^2^/s)				
10th Percentile	0.71 [0.05, 1.48]	1.26 [0.77, 1.91]	0.93 [0.87, 1.2]	0.001
90th Percentile	0.94 [0.15, 2.64]	1.62 [1.02, 2.16]	1.12 [1.06, 1.46]	0.016
Maximum	1.36 [0.28, 3.39]	1.71 [1.1, 2.21]	1.32 [1.2, 1.9]	0.217
Mean	0.81 [0.09, 2.08]	1.42 [0.88, 2.02]	1.03 [0.97, 1.31]	0.007
Median	0.8 [0.07, 2.1]	1.42 [0.87, 2.02]	1.03 [0.97, 1.31]	0.007
Minimum	0.52 [0, 1.11]	1.1 [0.51, 1.79]	0.8 [0.66, 0.94]	<0.001
Range	0.6 [0.18, 2.88]	0.45 [0.29, 0.75]	0.57 [0.38, 1.01]	0.240
IQR	0.14 [0.04, 0.87]	0.14 [0.09, 0.27]	0.11 [0.07, 0.22]	0.065
MAD	0.08 [0.03, 0.46]	0.08 [0.05, 0.16]	0.06 [0.05, 0.15]	0.039
RMAD	0.06 [0.02, 0.34]	0.06 [0.04, 0.12]	0.04 [0.03, 0.1]	0.056
RMS	0.81 [0.1, 2.12]	1.42 [0.88, 2.02]	1.03 [0.97, 1.31]	0.011
Entropy	7.07 [3, 11.12]	6.5 [4.89, 8.14]	4.3 [4, 5.35]	<0.001
Energy	87.49 [3.87, 45685.44]	276.59 [81.82, 1150.08]	1.58 [1.22, 2.81]	<0.001
Total Energy	785.62 [34.72, 410236.65]	2483.66 [734.74, 10327.23]	14183.41 [10985.3, 25261.8]	<0.001
Skewness	0.46 [−1.01, 2.45]	0.03 [−0.52, 0.56]	0.22 [−0.54, 1.2]	0.114
Kurtosis	3.34 [1.35, 11.62]	2.55 [1.98, 3.59]	3.27 [2.51, 5.4]	0.006
Uniformity	0.01 [0, 0.17]	0.01 [0, 0.03]	0.06 [0.03, 0.08]	<0.001
Variance	0.01 [0, 0.31]	0.01 [0, 0.04]	0.01 [0, 0.04]	0.034
FA-derived metrics				
10th Percentile	0.071 [0.024, 0.117]	0.073 [0.030, 0.606]	0.036 [0.029, 0.049]	<0.001
90th Percentile	0.171 [0.139, 0.282]	0.152 [0.081, 0.788]	0.091 [0.065, 0.128]	<0.001
Maximum	0.327 [0.154, 0.907]	0.239 [0.112, 0.888]	0.185 [0.100, 0.453]	0.002
Mean	0.117 [0.069, 0.183]	0.112 [0.061, 0.686]	0.062 [0.048, 0.083]	<0.001
Median	0.111 [0.051, 0.179]	0.107 [0.053, 0.679]	0.056 [0.047, 0.081]	<0.001
Minimum	0.042 [0.000, 0.087]	0.042 [0.009, 0.478]	0.013 [0.006, 0.021]	<0.001
Range	0.265 [0.093, 0.907]	0.199 [0.091, 0.410]	0.170 [0.093, 0.441]	0.03
IQR	0.059 [0.024, 0.107]	0.043 [0.025, 0.106]	0.026 [0.018, 0.047]	<0.001
MAD	0.038 [0.016, 0.069]	0.024 [0.014, 0.056]	0.017 [0.011, 0.031]	<0.001
RMAD	0.026 [0.010, 0.046]	0.018 [0.010, 0.043]	0.011 [0.007, 0.020]	<0.001
RMS	0.126 [0.086, 0.193]	0.117 [0.064, 0.690]	0.066 [0.050, 0.089]	<0.001
Entropy	3.431 [2.156, 4.370]	2.928 [2.182, 6.593]	2.452 [1.893, 3.051]	<0.001
Energy	3.353 [0.091, 368.614]	2.484 [0.458, 60.479]	6.068 [3.172, 11.431]	0.008
Total Energy	30.109 [0.818, 3310.003]	22.307 [4.115, 543.076]	54.485 [28.483, 102.642]	0.010
Skewness	0.959 [−0.243, 2.079]	0.513 [−0.139, 1.448]	1.135 [0.180, 3.864]	0.041
Kurtosis	3.751 [1.984, 10.922]	4.052 [1.962, 6.322]	5.722 [2.651, 31.354]	0.02
Uniformity	0.115 [0.058, 0.250]	0.156 [0.011, 0.259]	0.227 [0.134, 0.314]	<0.001
Variance	0.003 [0.000, 0.008]	0.001 [0.000, 0.005]	0.000 [0.000, 0.002]	<0.001

(ADC: apparent diffusion coefficient; FA: fractional anisotropy; IQR: interquartile range; MAD: mean absolute deviation; RMAD: robust mean absolute deviation; RMS: root mean square).

**Table 5:** Results of ordinal logistic regression analysis to predict tissue classification (group 1: TGCT, group 2: GCNIS, group 3: normal testes) using volumetric ADC and FA histogram metrics.

Variable	β [95% CI]	*p*
ADC-derived metrics		
10th Percentile	16.23 [0.55, 31.92]	0.042
Range	56.41 [7.78, 105.05]	0.023
IQR	−276.99 [−511.81, −42.17]	0.021
Entropy	−3.62 [−6.83,−0.41]	0.027
Total energy	3.52 × 10^−4^ [2.8 × 10^−5^, 0.001]	0.033
Skewness	6.25 [0.23, 12.27]	0.042
Kurtosis	−8.96 [−16.92, −1]	0.033
Uniformity	19.27 [−82.03, 120.58]	0.709
FA-derived metrics		
Median	39.17 [−5.11, 83.46]	0.083
Range	55.93 [−34.8, 146.67]	0.227
Energy	−0.29 [−0.57, −0.01]	0.039
Skewness	1.31 [−4.19, 6.82]	0.640
Kurtosis	0.01 [−2.29, 2.31]	0.995
Uniformity	73.64 [−38.77, 186.04]	0.199

(CI: confidence interval; IQR: interquartile range).

## Data Availability

The data that support the findings of this study are available from the corresponding author upon reasonable request.

## Abbreviations

The following abbreviations are used in this manuscript:

TGCTS	testicular germ cell tumors
GCNIS	Germ Cell Neoplasia In Situ
NSGCTS	non-seminomatous germ cell tumors
DWI	diffusion-weighted imaging
ADC	apparent diffusion coefficient
ROI	region of interest
3D	three-dimensional
DTI	diffusion tensor imaging
FA	fractional anisotropy
T2WI	T2-weighted imaging
DCE	dynamic contrast-enhanced
VOI	volume of interest
IQR	interquartile range
MAD	mean absolute deviation
RMAD	robust mean absolute deviation
RMS	root mean square
(H&E)	hematoxylin & eosin
OCT3/4	octamer-binding transcription factor 3/4
PLAP	placental alkaline phosphatase
ROC	Receiver Operating Characteristic
